# Genetic polymorphisms located in genes related to immune and inflammatory processes are associated with end-stage renal disease: a preliminary study

**DOI:** 10.1186/1471-2350-13-58

**Published:** 2012-07-20

**Authors:** Ma Angeles Jimenez-Sousa, Elisabeth López, Amanda Fernandez-Rodríguez, Eduardo Tamayo, Pablo Fernández-Navarro, Laura Segura-Roda, María Heredia, José I Gómez-Herreras, Jesús Bustamante, Juan Miguel García-Gómez, Jesús F Bermejo-Martin, Salvador Resino

**Affiliations:** 1 Unidad de Epidemiología Molecular de Enfermedades Infecciosas, Centro Nacional de Microbiología, Instituto de Salud Carlos III (Campus Majadahonda), Carretera Majadahonda- Pozuelo, Km 2.2, 28220, Majadahonda (Madrid), Spain; 2 Departamento de Anestesiología y Reanimación, Hospital Clínico Universitario, Valladolid, Spain; 3 Área de Epidemiología Ambiental y Cáncer Unit. Centro Nacional de Epidemiología, Instituto de Salud Carlos III, Madrid, Spain; 4 CIBER Epidemiología y Salud Pública (CIBERESP), Barcelona, Spain; 5 IBIME, Instituto de Aplicaciones de las Tecnologías de la Información y de las Comunicaciones Avanzadas (ITACA), Universitat Politècnica de València, València, Spain; 6 Departamento de Nefrología, Hospital Clínico Universitario, Valladolid, Spain; 7 Unidad de Investigación Médica en Infección e Inmunidad, Hospital Clínico Universitario-IECSCYL, Valladolid, Spain

**Keywords:** ESRD, Kidney, Genetic polymorphism, Inflammation, Immunity

## Abstract

**Background:**

Chronic kidney disease progression has been linked to pro-inflammatory cytokines and markers of inflammation. These markers are also elevated in end-stage renal disease (ESRD), which constitutes a serious public health problem.

**Objective:**

To investigate whether single nucleotide polymorphisms (SNPs) located in genes related to immune and inflammatory processes, could be associated with ESRD development.

**Design and methods:**

A retrospective case-control study was carried out on 276 patients with ESRD and 288 control subjects. Forty-eight SNPs were genotyped via SNPlex platform. Logistic regression was used to assess the relationship between each sigle polymorphism and the development of ESRD.

**Results:**

Four polymorphisms showed association with ESRD: rs1801275 in the *interleukin 4 receptor* (*IL4R*) gene (OR: 0.66 (95%CI = 0.46-0.95); *p* = 0.025; overdominant model), rs4586 in *chemokine (C-C motif) ligand 2* (*CCL2*) gene (OR: 0.70 (95%CI = 0.54-0.90); *p* = 0.005; additive model), rs301640 located in an intergenic binding site for *signal transducer and activator of transcription 4 (STAT4)* (OR: 1.82 (95%CI = 1.17-2.83); *p* = 0.006; additive model) and rs7830 in the *nitric oxide synthase 3* (*NOS3)* gene (OR: 1.31 (95%CI = 1.01-1.71); *p* = 0.043; additive model). After adjusting for multiple testing, results lost significance.

**Conclusion:**

Our preliminary data suggest that four genetic polymorphisms located in genes related to inflammation and immune processes could help to predict the risk of developing ESRD.

## Background

The prevalence of chronic kidney disease (CKD) and end-stage renal disease (ESRD) is growing worldwide, with the overall prevalence of CKD stages 3-5 being 6.83% in Spain [1]. Therefore, it constitutes a serious public health problem, which causes substantial morbidity and mortality [2]. Traditional risk factors for CKD progression include persistent proteinuria, dyslipidaemia, hypertension and smoking [3,4]. However, it has been postulated that non-traditional risk factors, such as oxidative stress, inflammation and immune processes, may also be important contributors to the pathogenesis of cardiovascular disease as well as progression to ESRD [5]. The inflammatory response involved in renal damage produces a release of proinflammatory cytokines and chemokines, which cause an increased inflow of leukocytes, intensification of interstitial nephritis and progressive fibrosis [6].

On the other hand, genetic susceptibility is also considered an important determining factor for the appearance and/or progression of ESRD and its complications [7,8]. Therefore, the study of polymorphisms may help us to look further into the disease pathogenesis and its underlying causes, as well as to predict the predisposition to developing the disease in order to identify the at-risk population. The aim of this study was to investigate whether single nucleotide polymorphisms (SNPs) located in regions affecting immune and inflammatory processes could be associated with development of ESRD. Our preliminary data suggest that four polymorphisms showed an association with ESRD protection or development, which could help to predict the risk of developing ESRD.

## Methods

### Patients

A retrospective study was carried out on 276 kidney transplant recipients (case-group) and 288 normal subjects (control-group) from the Hospital Clínico Universitario of Valladolid (Spain). The study was conducted in accordance with the Declaration of Helsinki. All patients and controls gave their written consent and the Institutional Ethics Committee approved the study.

Subjects in the case-group were patients older than 18 with ESRD that received a cadaver renal graft between December 1995 and October 2008. Subjects from the control-group were patients that underwent routine analysis at the hospital and had no evidence of renal pathology or other immune or inflammatory diseases. Individuals were selected to have age and gender distribution similar to the case-group patients at the time of transplantation. In this retrospective study, the clinical data and DNA samples were collected in a transversal way for both the case-group and the control-group between June 2008 and December 2008. In order to ensure homogeneity, all patients were Caucasian.

### DNA extraction, marker selection and genotyping

DNA was extracted from whole blood by the Chemagic Magnetic Separador Module1, CHEMAGEN® which uses a magnetic particles system to obtain DNA. The quantity of recovered DNA was quantified by using PicoGreen® dsDNA Quantitation Reagent (Molecular Probes, Inc., Eugene, Oregon, USA).

All SNPs were genotyped at the Spanish National Genotyping Centre (CeGen; http://www.cegen.org/) by the SNPlex genotyping system 48-plex (Applied Biosystems, Foster City, CA, USA) following the manufacturer’s recommended protocol (http://www3.appliedbiosystems.com/cms/groups/mcb_support/documents/generaldocuments/cms_042019.pdf). As quality control, two Centre d’Etude du Polymorphisme Humain (CEPH) samples (NA10860 and NA10861) from the HapMap database were included in all genotyping assays [9].

Published SNPs were selected by different criteria (see Additional file 1: Table S1 Content 1): SNPs associated with kidney disease or kidney complications in previous articles and/or SNPs located in genes involved in genetic pathways related to prevention or susceptibility to ESRD (inflammatory and immune processes),. With this purpose several databases were consulted: International HapMap Project (http://www.hapmap.org), dbSNP databases (http://www.ncbi.nlm.nih.gov/SNP/), as well as pathway databases such as GeneOntology (http://www.geneontology.org/) and KEGG (http://www.genome.jp/kegg/pathway.html). Besides, for each significant SNP, the biological implications were analyzed "in silico" via web-tools: SMART (http://smart.embl-heidelberg.de/) for identifying the protein domains, PSIPRED (http://bioinf.cs.ucl.ac.uk/psipred/) for analyzing protein secondary structure prediction, SIFT (http://blocks.fhcrc.org/sift/SIFT.html) for studying the amino acid change toleration, PATROCLES (http://www.patrocles.org/) for identifying putative microRNA binding sites, PROMO (http://alggen.lsi.upc.es/) for transcription factor binding site prediction and Human Splicing Finder v2.4.1 (http://www.umd.be/HSF/) for studying the pre-mRNA splicing sites.

### Statistical analysis

In the control group, Hardy-Weinberg equilibrium (HWE) was assessed for each polymorphism using the Pearson Chi-square (χ2) statistic. Only SNPs that observed HWE were included in association analysis.

Unconditional logistic regression was used to assess the relationship between each single polymorphism and the development of ESRD. Five genetic models were fitted (dominant, co-dominant, over-dominant, recessive, and log-additive model). For each SNP, likelihood ratio test (LRT), and Akaike's information criteria (AIC) were used as measures of the goodness of fit between models and to choose the inheritance model that best fits the data. The next equation defines the logistic model: log(p/1-p) = α + βG + yZ; with *p* being the probability, *G* the categorical variable with the polymorphisms codified, *Z* the variables to adjust the model (α, β and γ must be estimated). Odds ratios (OR) and 95% confidence intervals (CI) were calculated to check the relative risk for association. This model was adjusted for age and gender by SNPStats (http://bioinfo.iconcologia.net/SNPStats_web) [10] (p-value < 0.05 was considered significant). Moreover, multiple testing corrections were performed by SFDR (Stratified False Discovery Rate) software version 1.6. (http://www.utstat.utoronto.ca/sun/Software/SFDR/index.html).

## Results

Table 1 shows the clinical characteristics of all cases and controls. The median age and male female ratio were similar between groups. The most predominant primary disease involving renal failure was glomerulonephritis (≈30%).

**Table 1:** Clinical characteristics of patients with ESRD (cases) and control group

	**Case group**	**Control group**	**p value**
**No.**	276	288	
**Age**^**a**^	50.0 ± 0.78	52.8 ± 1.03	0.030
**Male**^**b**^	108 (39.1%)	119 (41.3%)	0.573
**Primary disease**^**b**^			
** Glomerulonephritis**	82 (29.7%)	NA	-
** Arterial hypertension**	30 (10.9%)	NA	-
** Diabetic nephropathy**	20 (7.2%)	NA	-
** Tubulointerstitial nephritis**	28 (10.1%)	NA	-
** Obstructive uropathy**	11 (4.0%)	NA	-
** Vascular causes**	5 (1.8%)	NA	-
** Polycystic kidney disease**	37 (13.4%)	NA	-
** Others**	63 (22.8%)	NA	-

^**a**^ Mean ± standard error of mean (s.e.m.). ^b^ Absolute number (percentage). NA: not applicable.

From 48 SNPs, the genotyping assay for six SNPs did not work (rs1570360, rs1808593, rs2070744, rs4762, rs5498, rs55634318); one SNP was monomorphic (rs175176); and five SNPs (rs1799969, rs1800471, rs1800871, rs4311, rs699) were discarded because they exceeded 10% of missing values. Therefore, we analyzed 36 SNPs, all of which fulfilled the minimum allele frequency (MAF) >0.05 for all samples and were in HWE in the control group.

Significant association with ESRD was found for 4 SNPs (Table 2): AG genotype of rs1801275 in *interleukin 4 receptor* (*IL4R*) showed reduced odds of ESRD assuming an overdominant model (adjusted OR = 0.66 (95%CI = 0.46-0.95), p = 0.025). In the case of rs4586 in *chemokine (C-C motif) ligand 2* (*CCL2*), the presence of each additional copy of the minor allele was associated with reduced odds of ESRD (adjusted OR = 0.70 (95%CI = 0.54-0.90), p = 0.005) in a log-additive model. On the other hand, rs301640 in an intergenic binding site for *signal transducer and activator of transcription 4 (STAT4)* and rs7830 in nitric oxide synthase 3 (*NOS3)* were associated with elevated odds of ESRD assuming a log-additive model (adjusted OR = 1.82 (95%CI = 1.17-2.83), p = 0.006; and adjusted OR = 1.31 (95%CI = 1.01-1.71), p = 0.043, respectively). After applying the false discovery rate (FDR) for multiple test correction, we obtained that adjusted p values for each hypothesis testing were no significant (q-value > 0.05). Allelic and genotypic frequencies of significant SNPs are showed in Additional file 2: Table S2 Content 2.

**Table 2:** Summary of logistic regression of SNPs with ESRD

**SNP**	**Gene**	**Inheritance model**	**Genotypes**	**OR (95**% **CI)**^**a**^	**p-value**^**a**^
**rs1801275**	*IL4R*	Overdominant	A/A-G/G	1.00	
			A/G	0.66 (0.46-0.95)	**0.025**
**rs301640**	*STAT4 binding site*	Log-additive	A	1.82 (1.17-2.83)	**0.0064**
**rs4586**	*CCL2*	Log-additive	C	0.70 (0.54-0.90)	**0.0051**
**rs7830**	*NOS3*	Log-additive	T	1.31 (1.01-1.71)	**0.043**

Abbreviations: ESRD, end-stage renal disease; IL4R, interleukin 4 receptor; STAT4 signal transducer and activator of transcription 4; CCL2, chemokine (C-C motif) ligand 2; NOS3 nitric oxide synthase 3; OR, odds ratio; 95%CI, 95% confidence interval; p-value, level of significance.

^a^OR and p-value of adjusted model by age and gender.

## Discussion

It has been previously shown that biomarkers of inflammation are high even in the early stages of CKD [11,12]. The increased inflammation could be caused by both genetic predisposition and environmental factors, being linked to the risk of CKD progression to ESRD. In our study, we have found strong suggestion of associations between ESRD and four SNPs located in *IL4R* (rs1801275), *CCL2* (rs4586), *NOS3* (rs7830), and an intergenic binding site for *STAT4* (rs301640). All of these sites involve genes related to inflammation and immune response pathways.

On the one hand, two of the significant SNPs (rs1801275, and rs4586) reflected a certain protection against ESRD:

a) rs1801275 (A/G) is located at position g.54150A > G of the *IL4R* gene in chromosome 16. This position corresponds to exon 12, where it generates a missense change (p.Gln576Arg). IL-4 is a cytokine involved in Th2 immune response, and its effect depends on binding with IL4R. IL-4 has been found to be an important predictor of kidney injury. Modulation of the IL4 pathway during ESRD may be due to an intracellular regulation involving different pathways but also could be possible that polymorphisms within the *IL4R* gene could alter the signalling pathway of IL-4, leading to a progression or prevention of kidney damage [13]. In our study, the presence of the AG genotype seems to indicate protection from ESRD development, which could be caused by a decrease in signalling of IL-4 through of its receptor. Although rs1801275 is a missense SNP which is located in a low complexity domain of the protein (SMART) and it has been predicted as tolerant (SIFT). To date, this SNP has shown associations with inflammatory and autoimmune diseases such as arthritis rheumatoid, asthma, allergic diseases and atopy [14,15], but the entire role in CKD of IL-4 and its signalling pathway through IL4R still remains unknown and further studies are needed.

b) rs4586 (C/T) is a synonymous polymorphism located on exon 2 of the *CCL2* gene in chromosome 17. The *CCL2* gene encodes a key chemokine in recruiting mononuclear inflammatory cells to sites of inflammation. One example is the interstitium and glomerulus, where CCL2 causes renal interstitial and glomerular inflammation, leading to progressive renal injury [16]. In our study, the presence of each additional copy of the C allele of rs4586 indicates protection against ESRD. Recent studies have suggested a beneficial effect of blocking the action of CCL2 on diabetic nephropathy and renal function through anti-fibrotic effects [17]. By analyzing the CCL2 sequence via PATROCLES, we have found that the C allele of rs4586 generates a putative target site (TGCTG**C**TA) for six different microRNAs (hsa-miR-15a/15b/16/195/424/497), whereas the T allele disrupt this target site and consequently none of these microRNAs target this sequence. MicroRNAs are small RNA molecules (22 nucleotides) that have a great impact on posttranscriptional regulation and potentially large relevance to complex diseases. When a microRNA attaches to its target, it can silence expression via mRNA degradation or by preventing mRNA translation [18]. Therefore we could hypothesize that C allele might exert its beneficial effect by binding a microRNA and blocking the transcription of CCL2 gene.

On the other hand, rs301640 and rs7830 polymorphisms indicated susceptibility to ESRD:

a) rs301640 (A/G) is a SNP located on chromosome 13, in an intergenic region between the *eukaryotic translation initiation factor 4A1 pseudogene 6* (EIF4A1P6) and the gene encoding hsa-miR-3169. This SNP is located within a binding site for the transcription factor STAT4. In our study, the presence of each additional copy of A allele was associated with increased odds of ESRD. By using PROMO software [19,20], we found that the A allele might disrupt the STAT4 binding site, which could potentially modify a distal enhancer. STAT4 plays an important role in Th1 differentiation by transmitting IL-12 signals to produce IFN-γ [21], which could induce pro-inflammatory cytokines leading to injury in target tissues.

b) rs7830 (G/T) is located in a region that belongs to two different genes. The sense strand corresponds to intron 26-27 of the *NOS3* gene, whereas the antisense strand matches with the 3´-UTR of the *autophagy related 9 homolog B* (*ATG9B*) gene. On the one hand, *NOS3* encodes for an enzyme that generates NO in endothelial cells and is involved in the regulation of vascular function [22]. On the other hand, *ATG9B* encodes for a protein required for autophagy in several eukaryotic organisms, although its entire function is unknown [23]. In our study, the T allele of rs7830 was associated with ESRD. We hypothesize that the effects of rs7830 on ESRD might be due to *NOS3* rather than *ATG9B*. In fact, polymorphisms in *NOS3* have been associated with atherosclerotic vascular diseases [24], renal dysfunction [25], and advanced diabetic nephropathy [26]. Moreover, this SNP seems to be located within a silencer motif (TGG**G**GACT) [27], where the G to T allele change could disturb the splicing mechanism in *NOS3* leading to different transcripts [28]. Thus, this change might affect *NOS3* expression and to be associated with the development of ESRD.

Although these findings have also been supported by previous studies where the SNPs described above have been associated with kidney disease or related (Additional file 1: Table S1 Content 1), we could not find any significant results after applying FDR correction. In regards to this, since p-value is depending on the sample size, it may be possible that we have not found any significant adjusted p-value because our sample size is not large enough to detect moderate effects. Thus only big effects would be detected in small populations. Moreover, it exists some controversy about adjusting the “p-value” after multiple tests on clinical-orientated studies [29,30]. In addition, the weak association found for the studied polymorphisms could be due to an indirect involvement in ESRD. That is, it cannot be discarded that these SNPs might be in linkage disequilibrium with more powerful polymorphisms associated with ESRD. Another limitation of our study was that although we tried to select individuals for control-group with similar age to case-group, the comparison between means was significant (50.0 versus 52.8 years; p = 0.030, Table 1). We think that these differences are so slight that have a low clinical significance. However, logistic regression analysis was adjusted by age in order to avoid possible age interferences.

Therefore, this is a preliminary study which could be considered to generate hypothesis for future studies. In fact, the association of these SNPs with ESRD needs to be confirmed by replicating studies with a larger sample size, as well as functional studies should be performed in order to get further insights into ESRD susceptibility. Moreover, it will be interesting to include in the future some factors which may also influence ESRD development (diabetes, dyslipidemia, smoking, etc.) which could not be collected in this study. Besides, those patients with ESRD who are receiving dialysis and/or have not yet received kidney transplant should also be taken into account in further studies because they could carry on different genotypes.

## Conclusions

In conclusion, our preliminary data suggest that four polymorphisms (rs1801275, rs301640, rs4586, rs7830) related to inflammatory and immunity processes showed an association with protection or development of ESRD. These results could help to predict the risk of developing the disease, and also to improve the understanding of the pathways involved in the disease pathogenesis.

## Abbreviations

AIC, Akaike’s information criteria; ATG9B, autophagy related 9 homolog B; CCL2, chemokine (C-C motif) ligand 2; CKD, chronic kidney disease; EIF4A1P6, eukaryotic translation initiation factor 4A1 pseudogene 6; ESRD, End-stage renal disease; FDR, false discovery rate; HWE, Hardy-Weinberg equilibrium; IL4R, interleukin 4 receptor; LRT, likelihood ratio test; NOS3, nitric oxide synthase 3; SNP, single nucleotide polymorphism; STAT4, signal transducer and activator of transcription 4.

## Competing interests

None of the authors has any potential financial conflict of interest related to this manuscript.

## Authors’ contribution

*Study concept and design:* ET, SR. *Administrative, technical, or material support:* EL, ET, MH, JIGH, JB, JFBM. *Acquisition of data:* EL, ET, MH, JIGH, JB. *Statistical analysis and interpretation of data:* MAJS, AFR, LSR, PF, JMGG, SR. *Drafting of the manuscript:* MAJS, AFR, SR. *Critical revision of the manuscript for important intellectual content:* PF, JFBM. *Study supervision:* SR. All authors read and approved the final manuscript.

## Pre-publication history

The pre-publication history for this paper can be accessed here:

http://www.biomedcentral.com/1471-2350/13/58/prepub

## Supplementary Material

Additional file 1 **Table S1. Content 1.** Description of gene polymorphisms included in the study. Click here for file

Additional file 2 ** Table S2. Content 2.** Allelic and genotypic frequencies of SNPs that showed association with ESRD. Click here for file
