# Supplementary material for: Genetic polymorphisms located in genes related to immune and inflammatory processes are associated with end-stage renal disease: a preliminary study
Source: BMC Med Genet. 2012 Jul 20;13:58. doi: 10.1186/1471-2350-13-58 (PMC3412707; doi:10.1186/1471-2350-13-58)
Supplement: Additional file 1 — Table S1. Content 1. Description of gene polymorphisms included in the study. [file 1471-2350-13-58-S1.doc]

**Supplemental Table Content 1. Description of gene polymorphisms included in the study**.

| **Gene** | **SNP ID** | **Change** | **Chr** | **Disease Association** |
| --- | --- | --- | --- | --- |
| Angiotensin I converting enzyme (ACE) | rs12449782 | A/G | 17 | Nephropathy (1, 2)  Cardiovascular diseases (3) |
|  | rs1800764 | C/T | 17 | Nephropathy (1, 2, 4) |
| Angiotensin II receptor, type 1 (AGTR1) | rs5186 | A/C | 3 | Chronic kidney failure (5) |
| Chemokine (C-C motif) ligand 5 (CCL5) | rs1800825 | C/T | 17 | Chronic graft-versus-host disease (6) |
|  | rs2107538 | C/T | 17 | Type 1 diabetes (7, 8)  Chronic graft-versus-host disease (6) |
| Chemokine (C-C motif) ligand 2 (CCL2) | rs4586 | C/T | 17 | Cardiovascular disease (9)  Inflammation and infection (10) |
| Interleukin 1, beta (IL1B) | rs1143634 | C/T | 2 | Cardiovascular diseases (11)  Obesity and metabolic syndrome (12)  Type 2 diabetes (13) |
| Interleukin 1 receptor antagonist (IL1RN) | rs419598 | C/T | 2 | Cardiovascular disease (14)  Inflammation (15) |
|  | rs2234676 | A/G | 2 | Inflammation and allergy (16) |
| Interleukin 4 (IL4) | rs2070874 | C/T | 5 | Inflammation and autoimmune disorders (17) |
|  | rs2243248 | G/T | 5 | Inflammation and autoimmune disorders (18) |
| Interleukin 4 receptor (IL4R) | rs1801275 | A/G | 16 | Inflammation and autoimmune disorders (19, 20) |
| Interleukin 6 (IL6) | rs1800795 | C/G | 7 | Chronic kidney failure (21)  Nephropathy (22)  Type 2 diabetes (23)  Cardiovascular diseases (23-25) |
|  | rs1800796 | C/G | 7 | Chronic kidney failure (21)  Type 2 diabetes and obesity (21)  Nephropathy (26, 27)  Cardiovascular disease (11, 25) |
|  | rs1800797 | A/G | 7 | Cardiovascular disease (25)  Metabolic syndrome (28) |
| Interleukin 10 (IL10) | rs1800872 | A/C | 1 | Type 2 diabetes (29)  Cardiovascular disease (11) |
|  | rs1800896 | A/G | 1 | Type 2 diabetes (29)  Cardiovascular disease (30) |
| Interferon, gamma (IFNG) | rs2430561 | A/T | 12 | Kidney transplant failure (31)  Nephropathy (32)  Type 1 diabetes (33)  Autoimmune disorders (34) |
| Matrix metallopeptidase 1 (MMP1) | rs1799750 | -/G | 11 | Chronic kidney failure (35)  Cardiovascular disease(36) |
|  | rs2071231 | G/T | 11 | Endometrial cancer risk (37) |
|  | rs470206 | A/G | 11 | Endometrial cancer risk (37) |
| Matrix metallopeptidase 2 (MMP2) | rs243865 | C/T | 16 | Cardiovascular disease (38)  Inflammation (39) |
| Intergenic region - STAT4 binding site | rs301640 | A/G | 13 | Inflammation (40) |
| Nitric oxide synthase 3 (NOS3) | rs3918226 | C/T | 7 | Inflammation (41)  Cardiovascular diseases (42) |
|  | rs7830 | G/T | 7 | Cardiovascular diseases (42) |
| Hepatitis A virus cellular receptor 1 (HAVCR1) | rs41297579 | A/G | 5 | Inflammation and infection (43) |
| Hepatitis A virus cellular receptor 2 (HAVCR2) | rs1036199 | A/C | 5 | Type 1 diabetes (44) |
|  | rs10515746 | A/C | 5 | Type 1 diabetes (44) |
| TIMP metallopeptidase inhibitor 3 (TIMP3) | rs5749511 | C/T | 22 | Cardiovascular disease (45) |
| Toll-like receptor 2 (TLR2) | rs5743708 | A/G | 4 | Inflammation and autoimmune disorders (46) |
|  | rs4696480 | A/T | 4 | Inflammation (47) |
| Toll-like receptor 4 (TLR4) | rs4986790 | A/G | 9 | Type 2 diabetes and metabolic syndrome (48) (49)  Cardiovascular disease (50-52) |
|  | rs4986791 | C/T | 9 | Type 2 diabetes (49) |
| Tumor necrosis factor (TNF) | rs1800629 | A/G | 6 | Kidney transplant failure (53, 54)  Type 1 diabetes (55)  Inflammation and autoimmune disorders (56, 57)  Metabolic syndrome (28) |
| Vascular endothelial growth factor A (VEGFA) | rs699947 | A/C | 6 | Cardiovascular disease (58)  Chronic graft-versus-host disease (59) |
|  | rs833061 | C/T | 6 | Chronic graft-versus-host disease (59) |

**REFERENCES**

1 Ezzidi I, Mtiraoui N, Kacem M, et al. Identification of specific angiotensin-converting enzyme variants and haplotypes that confer risk and protection against type 2 diabetic nephropathy. *Diabetes Metab Res Rev*. 2009;25:717-724.

2 Hadjadj S, Tarnow L, Forsblom C, et al. Association between angiotensin-converting enzyme gene polymorphisms and diabetic nephropathy: case-control, haplotype, and family-based study in three European populations. *J Am Soc Nephrol*. 2007;18:1284-1291.

3 Kulminski AM, Culminskaya IV, Ukraintseva SV, et al. Polymorphisms in the ACE and ADRB2 genes and risks of aging-associated phenotypes: the case of myocardial infarction. *Rejuvenation Res*. 2010;13:13-21.

4 Boright AP, Paterson AD, Mirea L, et al. Genetic variation at the ACE gene is associated with persistent microalbuminuria and severe nephropathy in type 1 diabetes: the DCCT/EDIC Genetics Study. *Diabetes*. 2005;54:1238-1244.

5 Lee YT, Chiu HC, Huang CT, et al. The A1166C polymorphism of angiotensin II Type 1 receptor as a predictor of renal function decline over 4 years follow-up in an apparently healthy Chinese population. *Clin Nephrol*. 2009;72:457-467.

6 Kim DH, Jung HD, Lee NY, Sohn SK. Single nucleotide polymorphism of CC chemokine ligand 5 promoter gene in recipients may predict the risk of chronic graft-versus-host disease and its severity after allogeneic transplantation. *Transplantation*. 2007;84:917-925.

7 Jeong KH, Moon JY, Chung JH, Kim YH, Lee TW. Significant associations between CCL5 gene polymorphisms and post-transplantational diabetes mellitus in Korean renal allograft recipients. *Am J Nephrol*. 2010;32:356-361.

8 Zhernakova A, Alizadeh BZ, Eerligh P, et al. Genetic variants of RANTES are associated with serum RANTES level and protection for type 1 diabetes. *Genes Immun*. 2006;7:544-549.

9 Amoli MM, Salway F, Zeggini E, Ollier WE, Gonzalez-Gay MA. MCP-1 gene haplotype association in biopsy proven giant cell arteritis. *J Rheumatol*. 2005;32:507-510.

10 Loeffler J, Steffens M, Arlt EM, et al. Polymorphisms in the genes encoding chemokine receptor 5, interleukin-10, and monocyte chemoattractant protein 1 contribute to cytomegalovirus reactivation and disease after allogeneic stem cell transplantation. *J Clin Microbiol*. 2006;44:1847-1850.

11 Zee RY, Glynn RJ, Cheng S, et al. An evaluation of candidate genes of inflammation and thrombosis in relation to the risk of venous thromboembolism: The Women's Genome Health Study. *Circ Cardiovasc Genet*. 2009;2:57-62.

12 Carter KW, Hung J, Powell BL, et al. Association of Interleukin-1 gene polymorphisms with central obesity and metabolic syndrome in a coronary heart disease population. *Hum Genet*. 2008;124:199-206.

13 Luotola K, Pietila A, Zeller T, et al. Associations between Interleukin-1 (Il-1) Gene Variations or Il-1 Receptor Antagonist Levels and the Development of Type 2 Diabetes. *J Intern Med*. 2010;269:322-332.

14 Fragoso JM, Delgadillo H, Llorente L, et al. Interleukin 1 receptor antagonist polymorphisms are associated with the risk of developing acute coronary syndrome in Mexicans. *Immunol Lett*. 2010;133:106-111.

15 Attur M, Wang HY, Kraus VB, et al. Radiographic severity of knee osteoarthritis is conditional on interleukin 1 receptor antagonist gene variations. *Ann Rheum Dis*. 2010;69:856-861.

16 Pullat J, Fleischer R, Becker N, et al. Optimization of candidate-gene SNP-genotyping by flexible oligonucleotide microarrays; analyzing variations in immune regulator genes of hay-fever samples. *BMC Genomics*. 2007;8:282.

17 Balsa A, Del Amo J, Blanco F, et al. Prediction of functional impairment and remission in rheumatoid arthritis patients by biochemical variables and genetic polymorphisms. *Rheumatology (Oxford)*. 2010;49:458-466.

18 Khalilzadeh O, Anvari M, Momen-Heravi F, et al. Gene polymorphisms of interleukin-4, interleukin-10 and transforming growth factor-beta in Graves' disease. *Clin Exp Med*. 2010;10:123-128.

19 Loza MJ, McCall CE, Li L, et al. Assembly of inflammation-related genes for pathway-focused genetic analysis. *PLoS One*. 2007;2:e1035.

20 Burgos PI, Causey ZL, Tamhane A, et al. Association of IL4R single-nucleotide polymorphisms with rheumatoid nodules in African Americans with rheumatoid arthritis. *Arthritis Res Ther*. 2010;12:R75.

21 Ng DP, Nurbaya S, Ye SH, Krolewski AS. An IL-6 haplotype on human chromosome 7p21 confers risk for impaired renal function in type 2 diabetic patients. *Kidney Int*. 2008;74:521-527.

22 Mittal RD, Manchanda PK. Association of interleukin (IL)-4 intron-3 and IL-6 -174 G/C gene polymorphism with susceptibility to end-stage renal disease. *Immunogenetics*. 2007;59:159-165.

23 Palmer CN, Kimber CH, Doney AS, et al. Combined effect of inflammatory gene polymorphisms and the risk of ischemic stroke in a prospective cohort of subjects with type 2 diabetes: a Go-DARTS study. *Diabetes*. 2010;59:2945-2948.

24 Riikola A, Sipila K, Kahonen M, et al. Interleukin-6 promoter polymorphism and cardiovascular risk factors: the Health 2000 Survey. *Atherosclerosis*. 2009;207:466-470.

25 Maitra A, Shanker J, Dash D, et al. Polymorphisms in the IL6 gene in Asian Indian families with premature coronary artery disease--the Indian Atherosclerosis Research Study. *Thromb Haemost*. 2008;99:944-950.

26 Buraczynska M, Jozwiak L, Ksiazek P, Borowicz E, Mierzicki P. Interleukin-6 gene polymorphism and faster progression to end-stage renal failure in chronic glomerulonephritis. *Transl Res*. 2007;150:101-105.

27 Kitamura A, Hasegawa G, Obayashi H, et al. Interleukin-6 polymorphism (-634C/G) in the promotor region and the progression of diabetic nephropathy in type 2 diabetes. *Diabet Med*. 2002;19:1000-1005.

28 Phillips CM, Goumidi L, Bertrais S, et al. Additive effect of polymorphisms in the IL-6, LTA, and TNF-{alpha} genes and plasma fatty acid level modulate risk for the metabolic syndrome and its components. *J Clin Endocrinol Metab*. 2010;95:1386-1394.

29 Forte GI, Pilato G, Vaccarino L, et al. Risk profiles in type 2 diabetes (metabolic syndrome): integration of IL-10 polymorphisms and laboratory parameters to identify vascular damages related complications. *Curr Pharm Des*. 2010;16:898-903.

30 Heiskanen M, Kahonen M, Hurme M, et al. Polymorphism in the IL10 promoter region and early markers of atherosclerosis: the Cardiovascular Risk in Young Finns Study. *Atherosclerosis*. 2010;208:190-196.

31 Zibar L, Wagner J, Pavlinic D, et al. The relationship between interferon-gamma gene polymorphism and acute kidney allograft rejection. *Scand J Immunol*. 2011.

32 Schena FP, Cerullo G, Torres DD, et al. Role of interferon-gamma gene polymorphisms in susceptibility to IgA nephropathy: a family-based association study. *Eur J Hum Genet*. 2006;14:488-496.

33 Morris GA, Lowe CE, Cooper JD, et al. Polymorphism discovery and association analyses of the interferon genes in type 1 diabetes. *BMC Genet*. 2006;7:12.

34 Kim K, Cho SK, Sestak A, et al. Interferon-gamma gene polymorphisms associated with susceptibility to systemic lupus erythematosus. *Ann Rheum Dis*. 2010;69:1247-1250.

35 Yoshida T, Kato K, Fujimaki T, et al. Association of genetic variants with chronic kidney disease in Japanese individuals. *Clin J Am Soc Nephrol*. 2009;4:883-890.

36 Buss A, Pech K, Roelver S, et al. Functional polymorphisms in matrix metalloproteinases -1, -3, -9 and -12 in relation to cervical artery dissection. *BMC Neurol*. 2009;9:40.

37 Beeghly-Fadiel A, Xiang YB, Deming SL, et al. No association between matrix metalloproteinase (MMP)-1, MMP-3, and MMP-7 SNPs and endometrial cancer risk. *Cancer Epidemiol Biomarkers Prev*. 2009;18:1925-1928.

38 Lacchini R, Jacob-Ferreira AL, Luizon MR, et al. Common matrix metalloproteinase 2 gene haplotypes may modulate left ventricular remodelling in hypertensive patients. *J Hum Hypertens*. 2011.

39 Barlas IO, Sezgin M, Erdal ME, et al. Association of (-1,607) 1G/2G polymorphism of matrix metalloproteinase-1 gene with knee osteoarthritis in the Turkish population (knee osteoarthritis and MMPs gene polymorphisms). *Rheumatol Int*. 2009;29:383-388.

40 Wei L, Vahedi G, Sun HW, et al. Discrete roles of STAT4 and STAT6 transcription factors in tuning epigenetic modifications and transcription during T helper cell differentiation. *Immunity*. 2010;32:840-851.

41 Schurks M, Kurth T, Buring JE, Zee RY. A candidate gene association study of 77 polymorphisms in migraine. *J Pain*. 2009;10:759-766.

42 Kullo IJ, Greene MT, Boerwinkle E, et al. Association of polymorphisms in NOS3 with the ankle-brachial index in hypertensive adults. *Atherosclerosis*. 2008;196:905-912.

43 Nuchnoi P, Ohashi J, Kimura R, et al. Significant association between TIM1 promoter polymorphisms and protection against cerebral malaria in Thailand. *Ann Hum Genet*. 2008;72:327-336.

44 Bruck P, Ramos-Lopez E, Bartsch W, Bohme A, Badenhoop K. TIM-3 polymorphisms in type 1 diabetes families. *J Hum Genet*. 2008;53:559-564.

45 Armstrong C, Abilleira S, Sitzer M, Markus HS, Bevan S. Polymorphisms in MMP family and TIMP genes and carotid artery intima-media thickness. *Stroke*. 2007;38:2895-2899.

46 Tsui FW, Xi N, Rohekar S, et al. Toll-like receptor 2 variants are associated with acute reactive arthritis. *Arthritis Rheum*. 2008;58:3436-3438.

47 Kerkhof M, Postma DS, Brunekreef B, et al. Toll-like receptor 2 and 4 genes influence susceptibility to adverse effects of traffic-related air pollution on childhood asthma. *Thorax*. 2010;65:690-697.

48 Steinhardt AP, Aranguren F, Tellechea ML, et al. A functional nonsynonymous toll-like receptor 4 gene polymorphism is associated with metabolic syndrome, surrogates of insulin resistance, and syndromes of lipid accumulation. *Metabolism*. 2010;59:711-717.

49 Kolz M, Baumert J, Muller M, et al. Association between variations in the TLR4 gene and incident type 2 diabetes is modified by the ratio of total cholesterol to HDL-cholesterol. *BMC Med Genet*. 2008;9:9.

50 Ameziane N, Beillat T, Verpillat P, et al. Association of the Toll-like receptor 4 gene Asp299Gly polymorphism with acute coronary events. *Arterioscler Thromb Vasc Biol*. 2003;23:e61-64.

51 Holloway JW, Yang IA, Ye S. Variation in the toll-like receptor 4 gene and susceptibility to myocardial infarction. *Pharmacogenet Genomics*. 2005;15:15-21.

52 Kolek MJ, Carlquist JF, Muhlestein JB, et al. Toll-like receptor 4 gene Asp299Gly polymorphism is associated with reductions in vascular inflammation, angiographic coronary artery disease, and clinical diabetes. *Am Heart J*. 2004;148:1034-1040.

53 Mytilineos J, Laux G, Opelz G. Relevance of IL10, TGFbeta1, TNFalpha, and IL4Ralpha gene polymorphisms in kidney transplantation: a collaborative transplant study report. *Am J Transplant*. 2004;4:1684-1690.

54 Chen Y, Cicciarelli J, Pravica V, Hutchinson IV. Long-range linkage on chromosome 6p of VEGF, FKBP5, HLA and TNF alleles associated with transplant rejection. *Mol Immunol*. 2009;47:96-100.

55 Boraska V, Zeggini E, Groves CJ, et al. Family-based analysis of tumor necrosis factor and lymphotoxin-alpha tag polymorphisms with type 1 diabetes in the population of South Croatia. *Hum Immunol*. 2009;70:195-199.

56 Ferguson LR, Huebner C, Petermann I, et al. Single nucleotide polymorphism in the tumor necrosis factor-alpha gene affects inflammatory bowel diseases risk. *World J Gastroenterol*. 2008;14:4652-4661.

57 Maxwell JR, Potter C, Hyrich KL, et al. Association of the tumour necrosis factor-308 variant with differential response to anti-TNF agents in the treatment of rheumatoid arthritis. *Hum Mol Genet*. 2008;17:3532-3538.

58 Kangas-Kontio T, Tapanainen JM, Huikuri H, et al. Variation in the vascular endothelial growth factor gene, carotid intima-media thickness and the risk of acute myocardial infarction. *Scand J Clin Lab Invest*. 2009;69:335-343.

59 Kim DH, Lee NY, Lee MH, Sohn SK. Vascular endothelial growth factor gene polymorphisms may predict the risk of acute graft-versus-host disease following allogeneic transplantation: preventive effect of vascular endothelial growth factor gene on acute graft-versus-host disease. *Biol Blood Marrow Transplant*. 2008;14:1408-1416.
