# Supplementary material for: Genetic polymorphisms located in genes related to immune and inflammatory processes are associated with end-stage renal disease: a preliminary study
Source: BMC Med Genet. 2012 Jul 20;13:58. doi: 10.1186/1471-2350-13-58 (PMC3412707; doi:10.1186/1471-2350-13-58)
Supplement: Additional file 2 — Table S2. Content 2. Allelic and genotypic frequencies of SNPs that showed association with ESRD. [file 1471-2350-13-58-S2.doc]

**Table 2.** Allelic and genotypic frequencies of SNPs that showed association with ESRD.

| **SNPs** | **Frequencies** | **Controls** | **Cases** | **p-value** |
| --- | --- | --- | --- | --- |
| **rs1801275** | **A** | 206.5 (77.5) | 214.5 (80) | 0.496 |
| **(n=534)** | **G** | 59.5 (22.5) | 53.5 (20) |  |
|  | **AA** | 157 (59) | 177 (66) | 0.094 |
|  | **AG** | 99 (37.2) | 75 (28) | ***0.023*** |
|  | **GG** | 10 (3.8) | 16 (6) | 0.162 |
| **rs301640** | **A** | 255.5 (93.6) | 243 (89) | 0.058 |
| **(n=546)** | **G** | 17.5 (6.4) | 30 (11) |  |
|  | **AA** | 239 (87.5) | 216 (79.1) | ***0.008*** |
|  | **AG** | 33 (12.1) | 54 (19.8) | ***0.014*** |
|  | **GG** | 1 (0.4) | 3 (1.1) | 0.312 |
| **rs4586** | **T** | 146.5 (58.1) | 179.5 (66.5) | ***0.049*** |
| **(n=522)** | **C** | 105.5 (41.9) | 90.5 (33.5) |  |
|  | **TT** | 86 (34.1) | 119 (44.1) | ***0.020*** |
|  | **CT** | 121 (48) | 121 (44.8) | 0.464 |
|  | **CC** | 45 (17.9) | 30 (11.1) | ***0.028*** |
| **rs7830** | **G** | 206 (72.7) | 186.5 (67.6) | 0.177 |
| **(n=559)** | **T** | 77 (27.3) | 89.5 (32.4) |  |
|  | **GG** | 149 (52.6) | 124 (44.9) | 0.068 |
|  | **GT** | 114 (40.3) | 125 (45.3) | 0.233 |
|  | **TT** | 20 (7.1) | 27 (9.8) | 0.247 |

Values expressed as absolute count (percentage).

Abbreviations: ESRD, end-stage renal disease; p-value, level of significance.
